# *Idi1* and *Hmgcs2* Are Affected by Stretch in HL-1 Atrial Myocytes

**DOI:** 10.3390/ijms19124094

**Published:** 2018-12-18

**Authors:** Chih-Yuan Fang, Mien-Cheng Chen, Tzu-Hao Chang, Chia-Chen Wu, Jen-Ping Chang, Hsien-Da Huang, Wan-Chun Ho, Yi-Zhen Wang, Kuo-Li Pan, Yu-Sheng Lin, Yao-Kuang Huang, Chien-Jen Chen, Wei-Chieh Lee

**Affiliations:** 1 Division of Cardiology, Kaohsiung Chang Gung Memorial Hospital, Chang Gung University College of Medicine, Kaohsiung 83301, Taiwan; cyfang@seed.net.tw (C.-Y.F.); erin05201982@yahoo.com.tw (W.-C.H.); cat206206@gmail.com (Y.-Z.W.); cjchen@adm.cgmh.org.tw (C.-J.C.); leeweichieh@yahoo.com.tw (W.-C.L.); 2 Graduate Institute of Biomedical Informatics, Taipei Medical University, Taipei 110, Taiwan; Kevinchang@tmu.edu.tw; 3 Division of Cardiovascular Surgery, Kaohsiung Chang Gung Memorial Hospital, Chang Gung University College of Medicine, Kaohsiung 83301, Taiwan; maxwu02@gmail.com (C.-C.W.); c9112772@ms25.hinet.net (J.-P.C.); 4 Institute of Bioinformatics and Systems Biology, National Chiao Tung University, Hsinchu 300, Taiwan; bryan@mail.nctu.edu.tw; 5 Division of Cardiology, Chang Gung Memorial Hospital, Chiayi 61363, Taiwan; q12070@adm.cgmh.org.tw (K.-L.P.); dissertlin@yahoo.com.tw (Y.-S.L.); 6 Department of Thoracic and Cardiovascular Surgery, Chang Gung Memorial Hospital, Chiayi 61363, Taiwan; yaokuang@gmail.com

**Keywords:** atrium, genes, lipid, myopathy

## Abstract

Background: Lipid expression is increased in the atrial myocytes of mitral regurgitation (MR) patients. This study aimed to investigate key regulatory genes and mechanisms of atrial lipotoxic myopathy in MR. Methods: The HL-1 atrial myocytes were subjected to uniaxial cyclic stretching for eight hours. Fatty acid metabolism, lipoprotein signaling, and cholesterol metabolism were analyzed by PCR assay (168 genes). Results: The stretched myocytes had significantly larger cell size and higher lipid expression than non-stretched myocytes (all *p* < 0.001). Fatty acid metabolism, lipoprotein signaling, and cholesterol metabolism in the myocytes were analyzed by PCR assay (168 genes). In comparison with their counterparts in non-stretched myocytes, seven genes in stretched monocytes (*Idi1*, *Olr1*, *Nr1h4*, *Fabp2*, *Prkag3*, *Slc27a5*, *Fabp6*) revealed differential upregulation with an altered fold change >1.5. Nine genes in stretched monocytes (*Apoa4*, *Hmgcs2*, *Apol8*, *Srebf1*, *Acsm4*, *Fabp1*, *Acox2*, *Acsl6*, *Gk*) revealed differential downregulation with an altered fold change <0.67. Canonical pathway analysis, using Ingenuity Pathway Analysis software, revealed that the only genes in the “superpathway of cholesterol biosynthesis” were *Idi1* (upregulated) and *Hmgcs2* (downregulated). The fraction of stretched myocytes expressing Nile red was significantly decreased by RNA interference of *Idi1* (*p* < 0.05) and was significantly decreased by plasmid transfection of *Hmgcs2* (*p* = 0.004). Conclusions: The *Idi1* and *Hmgcs2* genes have regulatory roles in atrial lipotoxic myopathy associated with atrial enlargement.

## 1. Introduction

Mitral regurgitation (MR) is an important valve disease related to heart failure [1]. Left atrial dilatation with myocardial stretch, due to MR, is a well-established predictor of poor prognosis in patients undergoing mitral valve repair or replacement. Because of its important role in the development of atrial fibrillation, left atrial dilatation can increase thromboembolic risk and accelerate the progression of heart failure [2]. Previous studies have reported the role of chronic atrial stretch in the development of atrial fibrillation [3,4]. Moreover, any structural change in the atrial myocardium (e.g., atrial myopathy) can cause major electrophysiological disturbances and endocardial remodeling, which can then induce thrombogenesis at the atrial endocardium [5,6].

The peroxisome proliferator-activated receptors (PPARs) are ligand-activated transcription factors that regulate genes that are important in fatty acid β-oxidation, and control the expression of fatty acid utilization genes by binding to specific promoter DNA response elements with its heterodimeric partner, the retinoid X receptor [7,8]. The family of PPARs comprises three isoforms: PPARα, PPARβ/δ, and PPARγ. Expression of PPARα is high in metabolically active tissues such as heart tissue. Expression of PPARα may have a role in fatty acid metabolism [9,10]. The myocardium utilizes fatty acids primarily for ATP production via mitochondrial fatty acid oxidation. However, altered expression of fatty acid oxidation enzymes can impair mitochondrial metabolism and lead to pathologic remodeling of the myocardium, typically through lipotoxicity, reactive oxidative stress, and ATP deficiency [11,12]. We recently reported differential gene expression related to left atrial structural remodeling in a pig model of MR [13]. Interestingly, the results for gene ontology and pathway enrichment analyses of the genes, expressed differentially in that study, revealed PPAR signaling in the KEGG pathway [13]. Additionally, expressions of genes linked to the PPAR signaling pathway, especially genes related to fatty acid β-oxidation, in the left atria of MR patients reportedly differs from patients with aortic valve disease and normal controls [14]. Mitral regurgitation patients also have significantly higher lipid expression of atrial myocytes compared to normal controls [14]. Perturbation of lipid homeostasis due to a fatty acid uptake–utilization mismatch in the heart reportedly leads to the development of lipotoxic cardiomyopathy, an acquired form of cardiomyopathy [12]. However, the key regulatory genes and mechanisms of atrial lipotoxic myopathy of MR have not been elucidated.

Uni-axial stretch is used for in situ observation of many cell types and to investigate mechanically-activated signaling pathways in various cells [15]. Volume overload during MR induces atrial myocyte stretching. Therefore, this study used the NST-140 cell stretching system to induce mechanical uni-axial cyclic stretching of HL-1 atrial myocytes to replicate the mechanical stretching of atrial myocytes of MR patients; and investigated fatty acid metabolism, lipoprotein signaling, cholesterol metabolism pathway-related regulatory genes, and lipid expression in the HL-1 atrial myocytes. This study aimed to investigate key regulatory genes and mechanisms of atrial lipotoxic myopathy in MR. Here, we found that *Idi1* and *Hmgcs2* genes revealed regulatory roles in atrial lipotoxic myopathy associated with atrial enlargement.

## 2. Results

### 2.1. Morphological Changes and Lipid Expression after Mechanical Stretching of HL-1 Atrial Myocytes

**Figure 1:**
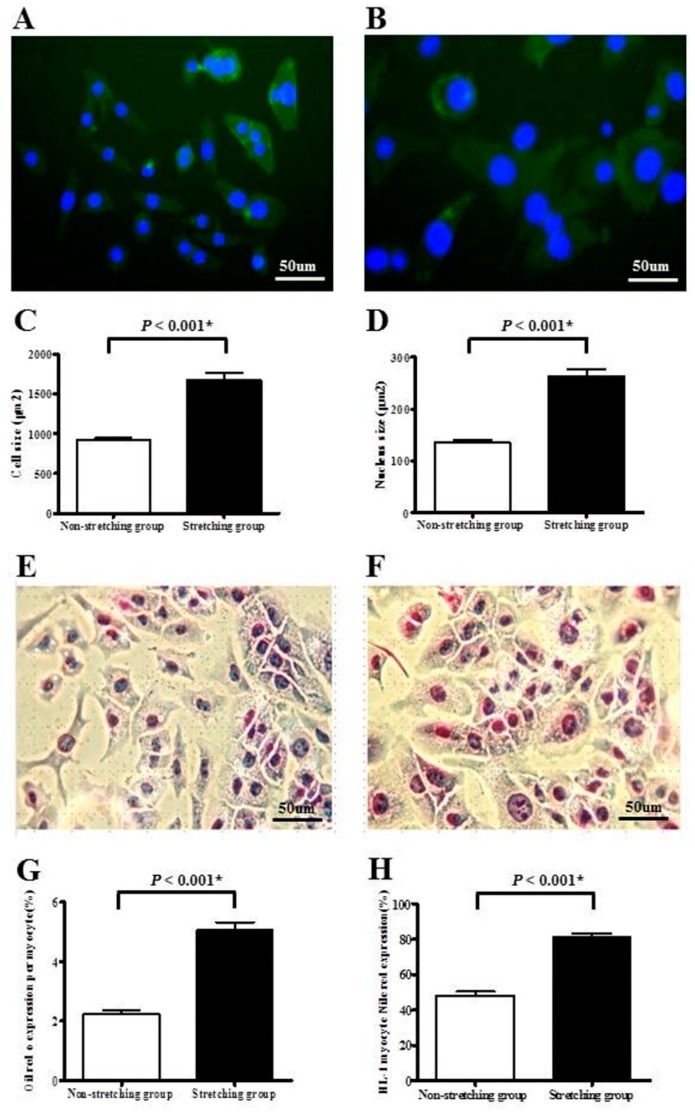
Immunofluorescence study of cell size and nucleus size of HL-1 atrial myocytes in non-stretched control group (**A**) and stretched group (**B**). Myocytes were identified with F-actin (green color). Nuclei were identified with DAPI (4′,6-diamidino-2-phenylindole) (blue color). Comparison of cell size (**C**) and nucleus size (**D**) between stretched and non-stretched HL-1 atrial myocytes. Histochemical analysis of lipid expression of HL-1 atrial myocytes in non-stretched control group (**E**) and stretched group (**F**). Comparison of Oil Red O-stained area per myocyte (**G**) and fraction of myocytes expressing Nile red (**H**), between the non-stretched control group and stretched group. * *p* < 0.05. Bar = 50 μm.

Figure 1 shows that, compared to non-stretched HL-1 atrial myocytes, stretched HL-1 atrial myocytes had significantly larger cell sizes (1681.4 ± 83.5 vs. 927.0 ± 22.0 µm^2^) and significantly larger nuclear sizes (263.2 ± 13.6 vs. 135.6 ± 4.2 µm^2^) (all *p* < 0.001). Stretched HL-1 atrial myocytes also had significantly higher lipid expression (Oil Red O-stained area/myocyte: 5.1% ± 0.2% vs. 2.3% ± 0.1%; the fraction of HL-1 atrial myocytes expressing Nile red: 80.9% ± 2.2% vs. 48.2% ± 2.1%) compared to non-stretched HL-1 atrial myocytes (Figure 1) (all *p* < 0.001).

**Figure 2:**
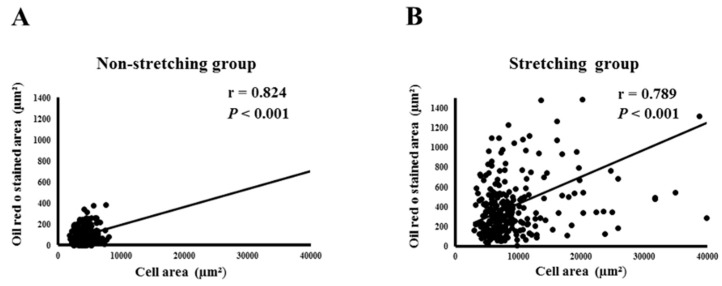
Correlation between cell size and Oil Red O-stained area of HL-1 atrial myocytes in the non-stretched control group (**A**) and stretched group (**B**).

Figure 2 shows the correlation analysis results, which revealed a significant direct association between the Oil Red O-stained area and cell area in both stretched (*r* = 0.789, *p* < 0.001) and non-stretched (*r* = 0.824, *p* < 0.001) HL-1 atrial myocytes. That is, lipid expression was significantly associated with atrial enlargement.

### 2.2. Gene Expression Analyses of Fatty Acid Metabolism, Lipoprotein Signaling, and Cholesterol Metabolism: Comparison of Stretched and Non-Stretched HL-1 Atrial Myocytes

**Figure 3:**
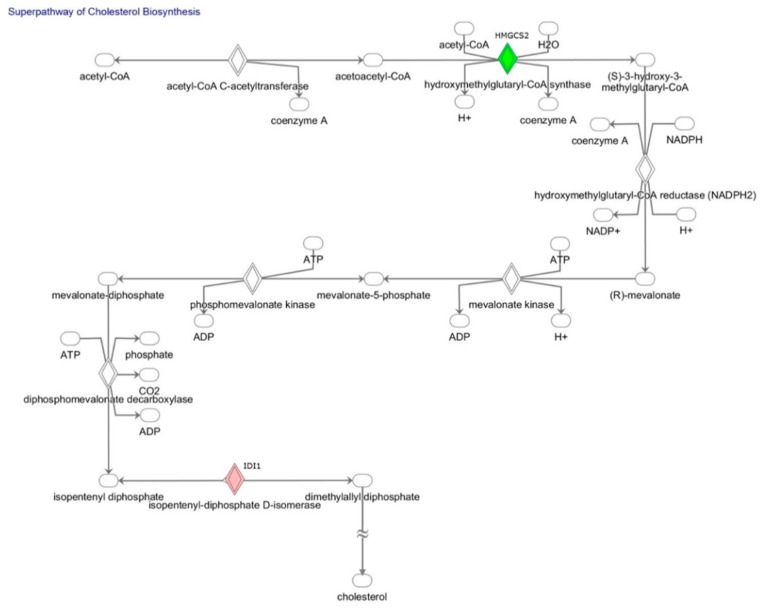
“Superpathway of cholesterol biosynthesis” in the Ingenuity Pathway Analysis. The only genes in the “superpathway of cholesterol biosynthesis” were *Idi1* and *Hmgcs2*.

This study used PCR assay to analyze and compare 168 genes with roles in the fatty acid metabolism, lipoprotein signaling, and cholesterol metabolism between stretched and non-stretched HL-1 atrial myocytes. Genes were ranked by fold change and/or significant difference (*p* < 0.05). Seven genes (*Idi1*, *Olr1*, *Nr1h4*, *Fabp2*, *Prkag3*, *Slc27a5*, *Fabp6*) revealed differential upregulation by fold change in altered genes >1.5 (Log_2_FC (stretched/non-stretched) >0.58). Nine genes (*Apoa4*, *Hmgcs2*, *Apol8*, *Srebf1*, *Acsm4*, *Fabp1*, *Acox2*, *Acsl6*, *Gk*) revealed differential downregulation by altered fold change <0.67 (Log_2_FC (stretched/non-stretched) <0.58), in stretched HL-1 atrial myocytes in comparison with non-stretched HL-1 atrial myocytes (Table 1). Figure 3 shows the canonical pathway analysis results obtained by Ingenuity Pathway Analysis software, which revealed that the only genes involved in “superpathway of cholesterol biosynthesis” were *Idi1* and *Hmgcs2* genes. Actually, all PCR-validated differentially-expressed genes were associated with gene ontology terms in fatty acid, lipoprotein, or cholesterol. However, canonical pathway analysis, using Ingenuity Pathway Analysis software, revealed two genes, *Idi1* and *Hmgcs2*, involved in the same pathway, because canonical pathways were curated and established according to experiment-validated results for the construction of regulatory direction between two genes. Therefore, the canonical pathway is more stringent, as compared with gene ontology terms, and may provide more biologically meaningful viewpoints.

### 2.3. Quantitative PCR Validation of Idi1 and Hmgcs2 mRNAs in the Stretched vs. Non-Stretched HL-1 Atrial Myocytes

**Figure 4:**
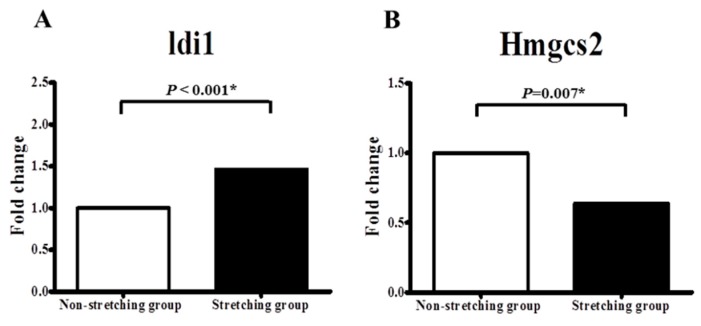
Quantitative determination of mRNAs of (**A**) isopentenyl-diphosphate delta isomerase (*Idi1*) and (**B**) 3-hydroxy-3-methylglutaryl coenzyme A synthase 2 (*Hmgcs2*), by real-time RT-PCR in non-stretched and stretched HL-1 atrial myocytes. * *p* < 0.05.

The expression of *Idi1* was significantly upregulated in stretched HL-1 atrial myocytes (*n* = 15) in comparison with non-stretched HL-1 atrial myocytes (*n* = 15) (0.49 ± 0.07 vs. 1.01 ± 0.11, *p* < 0.001) (Figure 4A). Whereas the expression of *Hmgcs2* was significantly downregulated in stretched HL-1 atrial myocytes in comparison with non-stretched HL-1 atrial myocytes (1.96 ± 0.26 vs. 1.03 ± 0.21, *p* = 0.007) (Figure 4B).

### 2.4. The Effect on Lipid Expression of Silencing the Idi1 Gene and Genetic Modification of the Hmgcs2 Gene, after Mechanical Stretching of HL-1 Atrial Myocytes

Canonical pathway analysis, using Ingenuity Pathway Analysis software, revealed that the only genes in “superpathway of cholesterol biosynthesis” were *Idi1* and *Hmgcs2*. Therefore, we analyzed the impacts of *Idi1* gene silencing and genetic modification of the *Hmgcs2* gene on the lipid expression of stretched HL-1 atrial myocytes.

**Figure 5:**
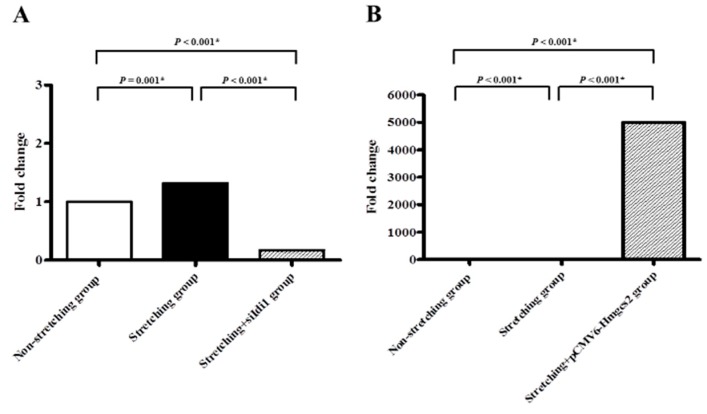
Quantitative determination of mRNAs by real-time RT-PCR in the HL-1 atrial myocytes. (**A**) Genetic silencing of *Idi1* mRNA; (**B**) genetic modification (plasmid transfection, pCMV6-*Hmgcs2*) of *Hmgcs2* mRNA. * *p* < 0.05.

**Figure 6:**
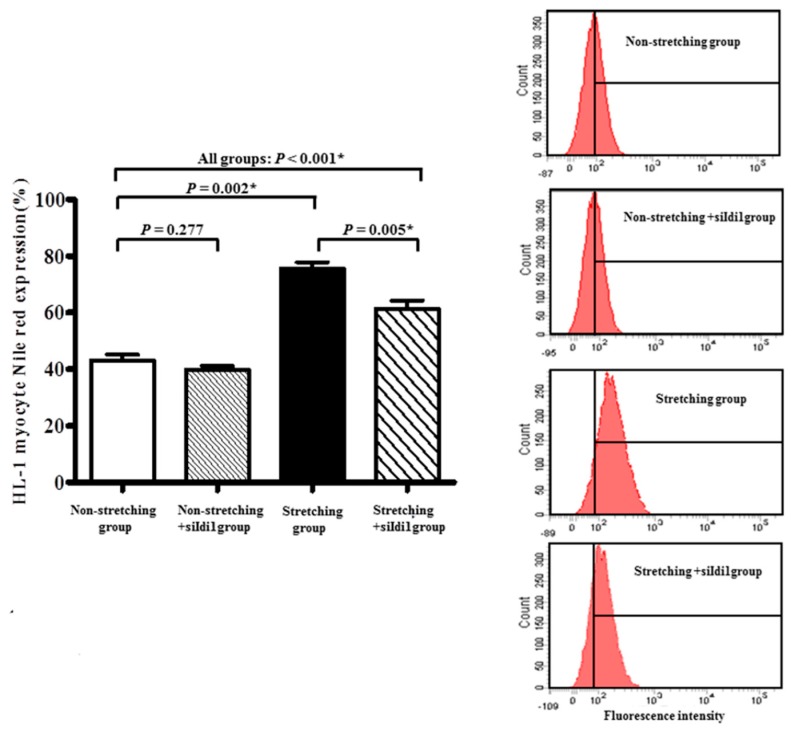
The fraction of HL-1 atrial myocytes expressing Nile red was determined by flow cytometry. Left panel: Comparison of fraction of HL-1 atrial myocytes expressing Nile red in non-stretched HL-1 atrial myocytes, non-stretched HL-1 atrial myocytes with genetic silencing of the *Idi1* mRNA, stretched HL-1 atrial myocytes, and stretched HL-1 atrial myocytes with genetic silencing of the *Idi1* mRNA. Right panel: Fluorescence intensity measurements for Nile red expression in non-stretched HL-1 atrial myocytes, non-stretched HL-1 atrial myocytes with genetic silencing of the *Idi1* mRNA, stretched HL-1 atrial myocytes, and stretched HL-1 atrial myocytes with genetic silencing of *Idi1* mRNA. * *p* < 0.05.

Genetic silencing of *Idi1* mRNA, which was significantly upregulated by stretching in the HL-1 atrial myocytes, was optimized by RNA interference. Expression of *Idi1* mRNA in stretched HL-1 atrial myocytes was significantly decreased by RNA interference of *Idi1* (0.77 ± 0.07 vs. 3.72 ± 0.11, *p* < 0.001) (Figure 5A). The percentage of stretched HL-1 atrial myocytes expressing Nile red was significantly decreased by RNA interference of *Idi1* (75.64% ± 2.24% vs. 61.44% ± 2.87%, *p* = 0.005) (Figure 6).

**Figure 7:**
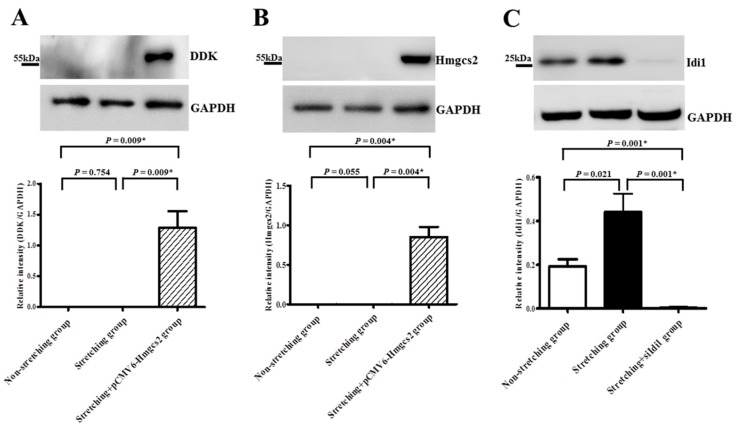
Immunoblot results for DDK (**A**), *Hmgcs2* (**B**), and *Idi1* (**C**) proteins in the non-stretched group, stretched group, and stretched group with plasmid transfection of pCMV6-*Hmgcs2* (Myc-DDK-tagged) (**A**,**B**), and the stretched group with RNA interference of *Idi1* (**C**). Plasmid transfection of pCMV6-*Hmgcs2* (Myc-DDK-tagged) in stretched HL-1 atrial myocytes was confirmed by significantly increased expressions of DDK (**A**) and *Hmgcs2* (**B**) in stretched HL-1 atrial myocytes that received plasmid transfection. Silencing of *Idi1* in stretched HL-1 atrial myocytes was confirmed by significantly decreased expression of *Idi1* (**C**) in stretched HL-1 atrial myocytes that had received RNA interference. * *p* < 0.05.

**Figure 8:**
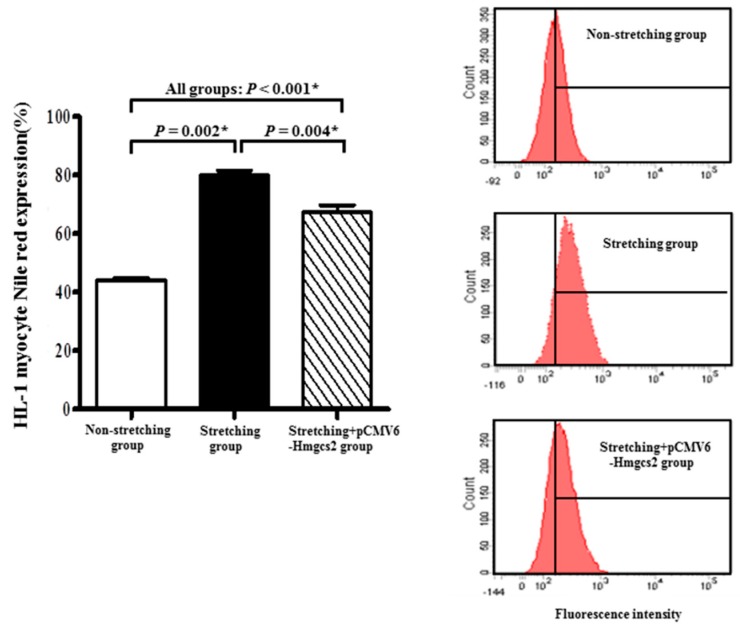
Left panel: Comparison of fraction of HL-1 atrial myocytes expressing Nile red in non-stretched HL-1 atrial myocytes, stretched HL-1 atrial myocytes, and stretched HL-1 atrial myocytes with genetic modification (plasmid transfection, pCMV6-*Hmgcs2*) of *Hmgcs2* mRNA. Right panel: Fluorescence intensity measurements for Nile red expression in non-stretched HL-1 atrial myocytes, stretched HL-1 atrial myocytes, and stretched HL-1 atrial myocytes with genetic modification of *Hmgcs2* mRNA. * *p* < 0.05.

Genetic modification (i.e., turned on) of the *Hmgcs2* mRNA, which was significantly downregulated by stretching in HL-1 atrial myocytes, was optimized by plasmid transfection (Figure 5B). Overexpression of transfected cell lysate for *Hmgcs2* (pCMV6-*Hmgcs2* (Myc-DDK-tagged)) was confirmed by Western blotting (Figure 7A). Expression of *Hmgcs2* mRNA in stretched HL-1 atrial myocytes was significantly increased by plasmid transfection of *Hmgcs2* (4.75 ± 0.15 vs. −9.51 ± 0.09, *p* < 0.001) (Figure 5B). The fraction of stretched HL-1 atrial myocytes expressing Nile red was significantly decreased by plasmid transfection of *Hmgcs2* (79.94% ± 1.72% vs. 67.31% ± 2.47%, *p* = 0.004) (Figure 8).

### 2.5. Immunoblot Analysis Confirmed Upregulation of Idi1 and Downregulation of Hmgcs2 by Stretching at the Translational Level

Expression of *Hmgcs2* protein was significantly downregulated in stretched HL-1 atrial myocytes (*n* = 6) in comparison with non-stretched HL-1 atrial myocytes (*n* = 6) (0.036 ± 0.011 vs. 0.083 ± 0.018, *p* = 0.055) (Figure 7B). Notably, *Hmgcs2* protein expression in stretched HL-1 atrial myocytes was significantly increased by plasmid transfection of *Hmgcs2* (0.036 ± 0.011 vs. 0.851 ± 0.127, *p* = 0.004) (Figure 7B).

Expression of *Idi1* protein was significantly upregulated in stretched HL-1 atrial myocytes (*n* = 8) in comparison with non-stretched HL-1 atrial myocytes (*n* = 8) (0.444 ± 0.081 vs. 0.19 ± 0.03, *p* = 0.021) (Figure 7C). Notably, *Idi1* protein expression in the stretched HL-1 atrial myocytes was significantly decreased by RNA interference of *Idi1* (0.444 ± 0.081 vs. 0.005 ± 0.002, *p* = 0.001) (Figure 7C).

## 3. Discussion

Stretching in HL-1 atrial myocytes altered the expression patterns of genes associated with fatty acid metabolism, lipoprotein signaling, and cholesterol metabolism. Notably, expressions of *Idi1*, *Olr1*, *Nr1h4*, *Fabp2*, *Prkag3*, *Slc27a5*, and *Fabp6* genes, associated with fatty acid metabolism and cholesterol metabolism, were significantly upregulated, whereas the expressions of *Hmgcs2*, *Apol8*, *Srebf1*, *Acox2*, *Acsl6*, and *Gk* genes were significantly downregulated in the stretched HL-1 atrial myocytes in comparison with non-stretched HL-1 atrial myocytes. Genetic silencing of the *Idi1* gene and genetic modification of the *Hmgcs2* gene reversed the lipid expression in the stretched HL-1 atrial myocytes.

Volume overload caused by MR induces atrial myocyte stretching, which then causes left atrial dilatation. In MR patients, altered mitochondrial function and reactive oxidative stress overproduction in the atria [16,17] altered fatty acid β-oxidation and lipid metabolism. Moreover, lipid expression of atrial myocytes in the left atria is significantly higher in MR patients compared to normal controls and compared to patients with aortic valve disease [14]. Furthermore, expressions of several genes in the PPAR pathway reveal differential expression in the left atria of MR patients in comparison with normal controls and patients with aortic valve disease [14]. In a pig model of MR in our study, gene ontology and pathway enrichment analysis in differentially-expressed genes revealed that the PPAR signaling pathway was identified in the KEGG pathway [13]. The PPAR transcriptional regulatory complex controls the expression of fatty acid utilization gene transcription for fatty acid oxidation [8]. In a novel mouse model of lipotoxic cardiomyopathy, Chiu et al. showed that a fatty acid uptake–utilization mismatch in the heart causes lipid accumulation, which is associated with initial cardiac hypertrophy, followed by the development of myocardial dysfunction and cardiac myocyte death, which is partly caused by lipid-induced programmed cell death [12]. In the advanced stages of heart failure, many key enzymes involved in myocardial energy substrate metabolism display various degrees of downregulation as a cardiac adaptation (metabolic switch), and the most notable change in the metabolic profile and altered metabolic phenotype of hypertrophied hearts consists of reduced cardiac fatty oxidation, increased glucose oxidation, and rigidity of the metabolic response to changes in workload [18,19,20]. In our study, mechanical stretching of HL-1 atrial myocytes, which mimicked the mechanical stretching of atrial myocytes in MR patients, resulted in significantly larger cell size and significantly higher lipid expression in stretched HL-1 atrial myocytes, in comparison with non-stretched HL-1 atrial myocytes. Mechanical stress has been implicated as one of the growth regulators in the heart and, consequently, the increase in nucleus size and cell size [21]. The increase in nuclear size indicated an increase in genome DNA content to generate larger cells, and that cell size was proportional to nuclear size, which was shown to reflect DNA content [22]. Stretched and non-stretched HL-1 atrial myocytes revealed differential expression in 13 of the 168 genes associated with fatty acid metabolism, lipoprotein signaling, and cholesterol metabolism. Moreover, canonical pathway analysis, performed using Ingenuity Pathway Analysis software, revealed that *Idi1* and *Hmgcs2* genes were the only genes involved in the “superpathway of cholesterol biosynthesis”. The important roles of the *Idi1* and *Hmgcs2* genes in the cholesterol biosynthesis pathway are well established [23,24]. Notably, reversing genetic expression of *Idi1* and *Hmgcs2* by genetic silencing and modification reversed lipid expression in stretched HL-1 atrial myocytes.

The *Idi1* (Isopentenyl-diphosphate delta isomerase 1) gene has a key role in the biosynthesis of isoprenoids through the mevalonate pathway, which results in synthesis of farnesyl diphosphate and, ultimately, synthesis of cholesterol [24]. Prior studies show that reactive oxygen species inhibit cellular expression of *Idi1* and block the formation of lipophilic molecules such as sterols, ubiquinones, and terpenoids [25]. Our study revealed that *Idi1* was significantly upregulated in stretched HL-1 atrial myocytes in comparison with non-stretched HL-1 atrial myocytes. Notably, *Idi1* mRNA expression in stretched HL-1 atrial myocytes was significantly decreased by RNA interference of *Idi1*. Therefore, Nile red expression of stretched HL-1 atrial myocytes was significantly decreased by RNA interference of *Idi1*.

The mitochondrial enzyme *Hmgcs2* (3-hydroxy-3-methylglutaryl-CoA synthase 2) induces fatty acid β-oxidation and ketogenesis in a metabolic pathway that provides lipid-derived energy [26]. Our study revealed significant downregulation of *Hmgcs2* expression in stretched HL-1 atrial myocytes in comparison with non-stretched HL-1 atrial myocytes. Notably, *Hmgcs2* mRNA expression in stretched HL-1 atrial myocytes was significantly increased by plasmid transfection of *Hmgcs2*, and Nile red expression of stretched HL-1 atrial myocytes was significantly decreased by plasmid transfection of *Hmgcs2* as a consequence of inducing fatty acid β-oxidation.

Taken together, the results of this study suggest that altered gene expression of fatty acid metabolism, lipoprotein signaling, and cholesterol metabolism might contribute to lipid accumulation and pathologic remodeling (hypertrophy) of HL-1 atrial myocytes in response to stretching. The results of this study may provide a rationale for metabolic therapies targeting genes expressed differentially in fatty acid and cholesterol metabolism, and/or targeting genes for posttranslational regulation to ameliorate atrial lipotoxic myopathy, associated with atrial dilatation and progression of heart failure in patients with MR.

## 4. Materials and Methods

### 4.1. Cell Culture and Mechanical Stretching of Atrial Myocytes

The HL-1 atrial myocytes, an immortalized mouse cardiomyocyte cell line while maintaining the phenotypic characteristics of the adult cardiomyocyte, were plated on silicone rubber culture dishes. The HL-1 atrial myocytes were cultured for 48 h in Claycomb medium containing 10% FBS, penicillin, and streptomycin. After 48 h, the culture medium was changed to serum-free Claycomb medium. The HL-1 atrial myocytes in the stretched group were subjected to 15% uniaxial cyclic stretching at 1 Hz for 8 h using an NST-140 cell stretching system (NEPA GENE, Chiba, Japan). Each experiment was performed simultaneously in the stretched and non-stretched control groups, with the same pool of HL-1 atrial myocytes with matching temperature, CO_2_ content, and pH of the medium.

### 4.2. Immunofluorescence Staining

To visualize cell size, the HL-1 atrial myocytes were stained with CytoPainter Phalloidin-iFluor 488 Reagent (Abcam, Cambridge, UK) (for staining F-actin) according to the manufacturer’s directions. Sections were mounted and visualized using a Dmi3000 microscope (Leica microsystems, Singapore). The F-actin- and DAPI (4′,6-diamidino-2-phenylindole, for staining nucleus)-stained areas per myocyte were analyzed by UTHSCSA, Image tool, Version 3.0 (Dental Diagnostic Science, San Antonio, TX, USA), with at least 50 randomly chosen myocytes in each experiment. Stretched and non-stretched myocytes were compared in seven different experiments.

### 4.3. Oil Red O Staining

To visualize lipid droplets, the HL-1 atrial myocytes were stained with Oil Red O (ScyTek Laboratories, Logan, UT, USA) according to the manufacturer’s directions. Sections were mounted and visualized with a Dmi3000 microscope. The Oil Red O-stained area per myocyte was analyzed by Cellsens Dimension (Olympus, Tokyo, Japan), with at least 60 randomly-chosen myocytes for each experiment. Stretched and non-stretched myocytes were compared in five experiments.

### 4.4. Measurement of Intracellular Lipid by Flow Cytometry

Nile red (Sigma Aldrich, St. Louis, MO, USA) was used to stain intracellular lipid. The HL-1 atrial myocytes were plated on silicone rubber culture dishes and cells were allowed to attach for 48 h. After 8 h of stretching, the cells were harvested by trypsinization, washed in PBS, and suspended in 5 μg/mL of Nile red. After incubation for 15 min at 40 °C, the cells were washed three times and suspended in PBS. In flow cytometry of intracellular Nile red content, 10,000 cells per sample were analyzed with a Cytomic FC500 (Beckman coulter, Indianapolis, IN, USA). The fraction of HL-1 atrial myocytes expressing Nile red was determined in the sorted 10,000 myocytes. Nile red fluorescence was analyzed at a wavelength of 530 ± 30 nm. A total of three experiments, comparing stretched and non-stretched myocytes, were performed. Each experiment was performed six times in each group.

### 4.5. PCR Assay and Data Processing

Extraction of RNA from HL-1 atrial myocytes (non-stretched group: *n* = 3; stretched group: *n* = 3) was performed with a Direct-zol^TM^ RNA MiniPrep kit (Zymo Reserach, Irvine, CA, USA) according to the manufacturer’s protocol. The PCR assay and data processing for fatty acid metabolism, lipoprotein signaling, and cholesterol metabolism related resources were obtained using information from RT^2^ profiler PCR array data analysis version 3.5 website (http://pcrdataanalysis.sabiosciences.com/pcr/arrayanalysis.php?target=upload). A total of 168 genes of the two pathways were examined by RT^2^ profiler PCR array (Qiagen, Redwood City, CA, USA) according to the manufacturer’s directions. Glucuronidase, beta (*Gusb*) gene served as the endogenous control. Fold-change values greater than one indicated a positive- or up-regulation, and fold-change values less than one indicated a negative- or down-regulation.

### 4.6. Quantitative Determination of RNAs by Real-Time PCR

The RNA samples were quantified with a spectrophotometer. First-strand cDNAs were synthesized with reverse transcriptase and oligo (dT) primers. Real-time quantitative PCR was performed with SYBR Green PCR Master Mix (Qiagen, CA, USA) on the ABI Prism 7500 FAST sequence detection system (Applied Biosystems, Foster City, CA, USA). The RNA results were normalized against *Gusb* gene expression (endogenous control). Three experiments were performed for each gene. Each experiment was repeated five times in each gene. Table 2 presents selected genes and primer sequences. Quantitative RT-PCR values are presented in ∆*Cq* units.

### 4.7. RNA Interference and In Vitro siRNA Transfection 

The siRNA sequences targeting the *Idi1* gene (National Center for Biotechnology Information (NCBI), accession number NM_145360) were synthesized by GenePharma (Shanghai, China). The HL-1 atrial myocytes were transfected with siRNAs using the lipofectamine RNAi MAX reagent protocol (Invitrogen, Carlsbad, CA, USA) one day before stretching. Confluence of cells was 50–60% on the day of transfection. The RNAi MAX reagent was diluted in opti-MEM medium. The HL-1 atrial myocytes were transfected with siRNA at a concentration of 30 pmol. Control cells without siRNA were incubated in medium with or without RNAi MAX reagent. The siRNA/RNAi MAX was added to cells grown in serum-free Claycomb medium. The cells were incubated for 8 h at 37 °C. The medium was then changed to 10% FBS Claycomb medium, and the cells were incubated for another 24 h. Next, the culture medium was changed to serum-free Claycomb medium, and HL-1 atrial myocytes were stretched for 8 h. For analysis of Nile red expression, three experiments were performed in the experimental group (*n* = 7) and in the control group (*n* = 7). For analysis of gene expression, three experiments were performed three times each in the experimental group and in the control group.

### 4.8. Plasmid Transfection

The pCMV6-*Hmgcs2* (Myc-DDK-tagged) was cloned by OriGene (Rockville, MD, USA) with Sgf I-Rsr II as restriction sites. The HL-1 atrial myocytes were transfected with lipofectamine 2000 reagent (Invitrogen, Carlsbad, CA, USA). The lipofectamine 2000 was added to cells grown in serum-free Claycomb medium and cells were incubated for 8 h at 37 °C. The cells were then incubated in 10% FBS Claycomb medium for 24 h. Next, the culture medium was changed to serum-free Claycomb medium, and the HL-1 atrial myocytes were stretched for 8 h. For analysis of Nile red expression, three experiments were performed in the experimental group (*n* = 7) and the control group (*n* = 7). For analysis of gene expression, three experiments were performed three times each in the experimental group and the control group.

### 4.9. Western Blot Analysis

The HL-1 myocytes were lysed in ice-cold RIPA lysis buffer. Protein concentrations in the lysates were determined by Pierce protein assay against bovine serum albumin standards. Cellular protein was separated in a 10% to 12% Tris-glycine gel and immunoblotted onto polyvinylidene difluoride membranes. The membranes were blocked with 5% non-fat milk in phosphate buffered saline with Tween 20 for 1 h and then incubated at 4 °C overnight with mouse anti-DDK antibody (OriGene, Rockville, MD, USA), rabbit anti-*Hmgcs2* antibody, and rabbit anti-*Idi1* antibody (Abcam, Cambridge, UK). Immunoreactivity was revealed with horseradish peroxidase-conjugated goat anti-mouse or donkey anti-rabbit antibody at room temperature. The protein assay results were normalized against glyceraldehyde-3-phosphate dehydrogenase (GAPDH).

### 4.10. Statistical Analysis

Data were presented as means ± SEM. In the non-stretched group, data for cell size, nucleus size, *Idi1*, and *Hmgcs2* were not normally distributed. In the stretched group, data for *Hmgcs2* were not normally distributed (*p* < 0.05 by Shapiro–Wilk test). Therefore, continuous variables between the two groups were analyzed by non-parametric test (i.e., Mann–Whitney Test). Pearson’s correlation was performed to determine the relationship between the cell size of the HL-1 atrial myocytes and the area of Oil Red O expression per myocyte. Statistical analysis was performed with commercially available statistics software (IBM SPSS Statistics 22). A *p* value (2-tailed) of <0.05 was considered statistically significant.

## 5. Conclusions

Genes associated with the fatty acid and cholesterol metabolism pathways were differentially expressed in stretched and non-stretched HL-1 myocytes. Two of the differentially expressed genes, *Idi1* and *Hmgcs2*, play regulatory roles in atrial lipotoxic myopathy, which is associated with atrial enlargement.

## Figures and Tables

**Table 1:** Selected gene expressions revealed by PCR assay for fatty acid metabolism, lipoprotein signaling, and cholesterol metabolism: Comparison of stretched and non-stretched HL-1 atrial myocytes.

Genes	Fold Change (Stretched/Non-Stretched)	*p* Value
**Upregulated (fold change > 1.5 and/or *p* < 0.05)**		
*Olr1*	2.8307	
*Nr1h4*	2.4049	
*Fabp2*	2.2812	
*Prkag3*	2.2569	
*Slc27a5*	1.7197	
*Fabp6*	1.5525	
*Idi1*	1.4388	<0.04
**Downregulated (fold change < 0.67)**		
*Apoa4*	0.3939	
*Hmgcs2*	0.4057	
*Acsm4*	0.4868	
*Apol8*	0.5564	
*Fabp1*	0.5858	
*Srebf1*	0.5859	
*Acox2*	0.649	
*Acsl6*	0.6512	
*Gk*	0.6589	

*Acox2* = Acyl-Coenzyme A oxidase 2, branched chain; *Acsl6* = Acyl-CoA synthetase long-chain family member 6; *Fabp2* = Fatty acid binding protein 2, intestinal; *Fabp6* = Fatty acid binding protein 6, ileal (gastrotropin); *Hmgcs2* = 3-hydroxy-3-methylglutaryl coenzyme A synthase 2; *Prkag3* = Protein kinase, AMP-activated, gamma 3 non-catatlytic subunit; *Slc27a5* = Solute carrier family 27 (fatty acid transporter), member 5; *Olr1* = Oxidized low density lipoprotein (lectin-like) receptor 1; *Nr1h4* = Nuclear receptor subfamily 1, group H, member 4; *Idi1* = Isopentenyl-diphosphate delta isomerase; *Apol8* = Apolipoprotein L 8; Srebf1 = Sterol regulatory element binding transcription factor 1; *Gk* = Glycerol kinase; *Apoa4* = Apolipoprotein A-IV; *Acsm4* = Acyl-CoA synthetase medium-chain family member 4; *Fabp1* = Fatty acid binding protein 1, liver.

**Table 2:** Primer sequences for real-time PCR.

Gene	Forward Primer	Reverse Primer
*Hmgcs2*	CAGGAAACTTCGCTCACACC	GGAGCAGGAGGGATTGTAGA
*Idi1*	CGAGCGATTGGATATGCTG	AATGTCTGATCTGACCTAGAACACAG
*Gusb*	GATGTGGTCTGTGGCCAAT	TGTGGGTGATCAGCGTCTT

*Hmgcs2* = 3-hydroxy-3-methylglutaryl Coenzyme A synthase 2; *Idi1* = Isopentenyl-diphosphate delta isomerase; *Gusb* = Glucuronidase, beta.
